# Magnetism and high magnetic-field-induced stability of alloy carbides in Fe-based materials

**DOI:** 10.1038/s41598-018-20910-3

**Published:** 2018-02-14

**Authors:** T. P. Hou, K. M. Wu, W. M. Liu, M. J. Peet, C. N. Hulme-Smith, L. Guo, L. Zhuang

**Affiliations:** 1 The State Key Laboratory for Refractories and Metallurgy, Hubei Province Key Laboratory of Systems Science in Metallurgical Process, International Research Institute for Steel Technology, Wuhan University of Science and Technology, Wuhan, 430081 China; 2 Beijing National Laboratory for Condensed Matter Physics, Institute of Physics, Chinese Academy of Sciences, Beijing, 100190 China; 3 Department of Materials Science and Metallurgy, University of Cambridge, Cambridge, UK; 4 Materials department, Centre of Excellence for Advanced Materials, Dongguan, 523808 China; 5 Sun Yat-Sen University, Guangzhou, 510275 China

## Abstract

Understanding the nature of the magnetic-field-induced precipitation behaviors represents a major step forward towards unravelling the real nature of interesting phenomena in Fe-based alloys and especially towards solving the key materials problem for the development of fusion energy. Experimental results indicate that the applied high magnetic field effectively promotes the precipitation of M_23_C_6_ carbides. We build an integrated method, which breaks through the limitations of zero temperature and zero external field, to concentrate on the dependence of the stability induced by the magnetic effect, excluding the thermal effect. We investigate the intimate relationship between the external field and the origins of various magnetics structural characteristics, which are derived from the interactions among the various Wyckoff sites of iron atoms, antiparallel spin of chromium and Fe-C bond distances. The high-magnetic-field-induced exchange coupling increases with the strength of the external field, which then causes an increase in the parallel magnetic moment. The stability of the alloy carbide M_23_C_6_ is more dependent on external field effects than thermal effects, whereas that of M_2_C, M_3_C and M_7_C_3_ is mainly determined by thermal effects.

## Introduction

Magnetic-field-induced carbide precipitation behaviours are a primary scientific and technological issue under the extreme operational conditions of a high magnetic field and intermediate temperature (300~550 °C, 3~4 Tesla) in International Thermonuclear Experimental Reactor (ITER)^1,2^. In the traditional heat treatment, alloy carbide M_23_C_6_ (M = Cr, Fe, Mn or Mo etc.) with a rich flexible character can be found in higher temperature (>600 °C) during the late tempering stage (about 1000 h) as a sphere or rod-like particle within laths or at the lath boundaries^3–6^. It is to be borne in mind that alloy carbides are usually in the paramagnetic state at the service temperature, and the external field has almost no influence on them^7,8^. However, experimental observations have also proven that the precipitation sequence, substitutional solute atom concentration and growth behaviours of iron and alloy carbides are influenced by high magnetic fields at lower temperature for a much shorter time^9–13^. It reveals that high-magnetic-field has a great effect on the stability of alloy carbides.

The initial work related to the magnetic-induced-stability was discussed qualitatively by means of the relationship between the carbon content and the magnetic moment at absolute temperature for iron carbide χ-Fe_5_C_2_^10^ and (Fe, Mo)_6_C^12^. More recent work has demonstrated magnetism has a determining influence on the stability of carbide with^14^ or without the external field^15^. The replacement of Mo^16^ or Cr atoms^17^ by iron influences the magnetism of the carbides, and then decreases their stability. The magnetic moment, as the optimal solution to the classical Weiss molecular field theory, has been long understood to be a fixed parameter^18,19^. However, the magnetism evolution exhibits a rich variety that are dependent on the complex structure^20^ and the coupling of the magnetic field and temperature^21,22^. Therefore, magnetic-field-induced stability needs to be triggered with external field intensity, various magnetism characteristics and magnetic-field-induced exchange coupling for different types of carbides at the various temperatures.

Here, we investigate the effect of a high magnetic field on the precipitation behavior by means of transmission electron microscopy and a hybrid method combining the thermodynamic equilibrium software MTDATA^14,23^, first-principles calculation as well as Weiss molecular field theory. We first investigate an intimate relationship of the external field and the origins of various magnetism characteristics which are derived from the interactions among the various Wyckoff sites of iron atoms, antiparallel spin of chromium and Fe-C bond distances. The high-magnetic-field-induced exchange coupling increases with the strength of the external field, which then causes an increase in the parallel magnetic moment. We demonstrate that the magnetic field has a predominant effect during the whole temperature range for (Fe, Cr)_23_C_6_ which have the larger magnetic moment, whereas thermal effects are much more significant for (Fe, Cr)_7_C_3_, Fe_3_C and Fe_2_C. The best agreement with the experiment is obtained when considering the interactions of the magnetism characteristics and magnetic-field-induced stability. These findings contribute to a better understanding of the creep-resistant property in reduced activation steels and effectively illuminate the key magnetism problem in the magnetic confinement Tokamak of the well-known ITER project and the other Fe-based alloys and compounds in extreme conditions.

## Results

### Precipitation stability of alloy carbides under the high magnetic field

The raw materials and the heat treatment procedures have been described in detail in previous publications^11,24^. Iron and alloy elements (Cr, Mo, V, W, Ti, etc.) are easily combined with C to form pure iron carbide and alloy carbide, respectively. At lower temperature, iron carbide preferentially precipitates. Consequently, higher temperatures are needed for the necessary diffusion of the alloying elements into iron carbide to form alloy carbide, which is thermodynamically more stable than pure iron carbide. We specify “the thermal effect” which means that the Gibbs free energy dependent with certain chemical composition and temperature without an external field. The thermal effect which is crucial for understanding of creep resistance properties, correlates with the d-elements filling, W or Mo impurities^22^, and Fe concentration^25,26^. Thermal effect is understood by means of the traditional thermodynamic equilibrium assessment^23,27^, as well as the formation energy at T = 0 K^4,22^, the vibration contribution^28,29^ and especially the magnon-phonon coupling at the formation temperature^15,30^.

**Figure 1:**
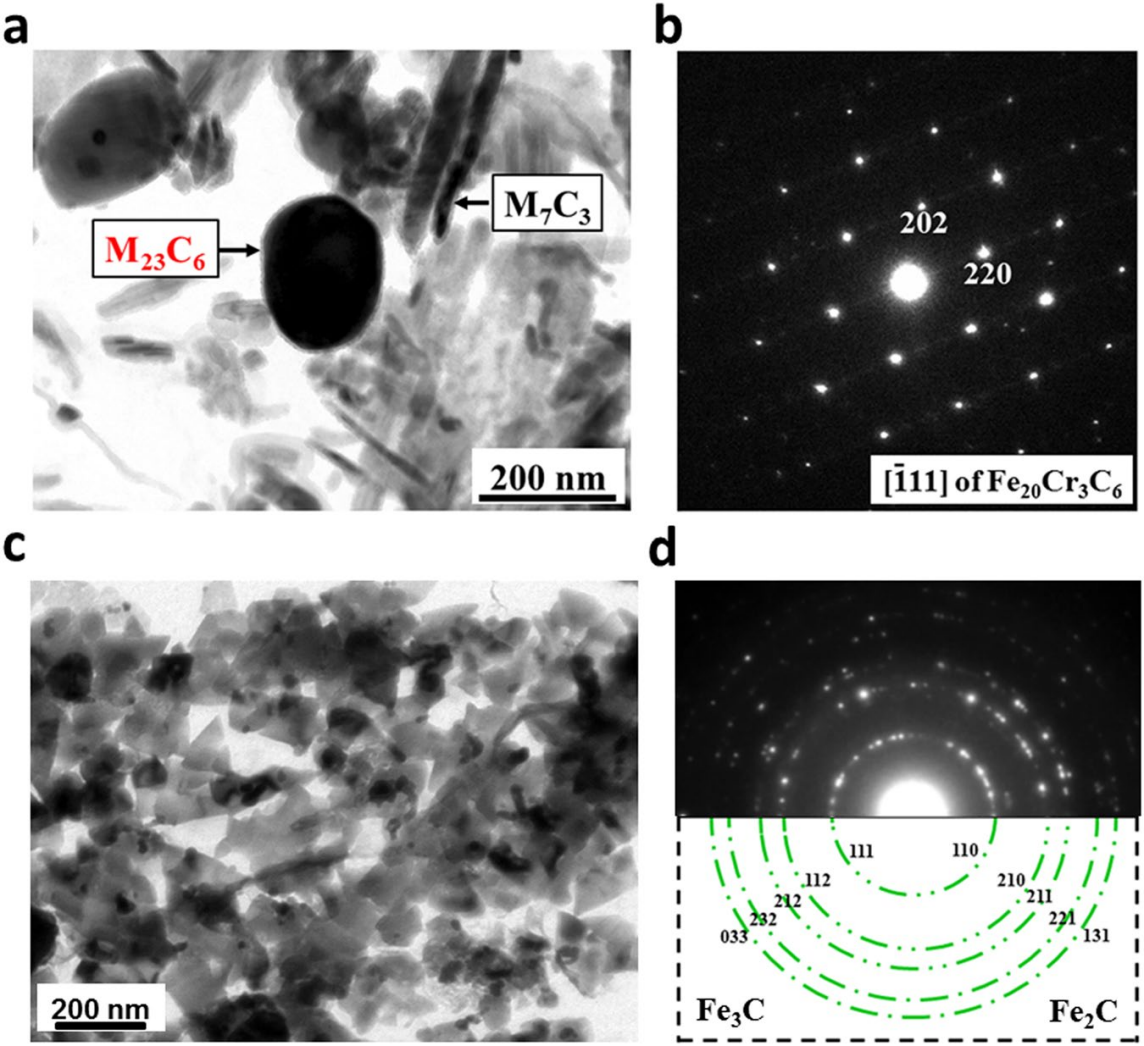
Transmission electron microscopy micrographs of four carbides: M_23_C_6_, M_7_C_3_, M_3_C, and M_2_C. Morphology (**a**,**c**) and selected area electron diffraction (SAED) (**b**,**d**) of Fe_19.51_Cr_3.49_C_6_, M_7_C_3_, M_3_C and M_2_C carbides during tempering at 823 K for 3600 s in the presence of a 12 T magnetic field. The measured aspect ratio for ellipsoid M_23_C_6_ is almost independent of whether the external field is applied or not^25^. For M_23_C_6_ in Fig. 1b, the spots that satisfy the condition that h, k, and l are all odd or all even are identified by means of SAED patterns. The characteristic faulted structure of M_7_C_3_ particles makes them easy to distinguish from particles of other carbides^34^. In Fig. 1d, the same diffraction ring within experimental errors corresponds to different crystal planes due to the similar d-spacing for orthorhombic M_3_C and M_2_C.

In the traditional heat treatment, M_23_C_6_ (M = Cr, Fe, Mn or Mo etc.) with a rich flexible character can be found at the higher temperature (>600 °C) during the late tempering stage (about 1000 h)^3,6^. However, the precipitation sequence of alloy carbides is significantly influenced by a 12 Tesla high magnetic field at the lower temperature (550 °C) for much shorter time (1 h)^24^. In the present work, the specimens in 2.25Cr-Mo steel were heat treated at 550 °C for 3600 s with and without a 12-T magnetic field. Figure 1a,b show the morphology and selected area electron diffraction (SAED) patterns of M_23_C_6_ and M_7_C_3_^31^ at a tempering temperature of 823 K for 3600 s with high magnetic field. The carbides M_23_C_6_ with space group Fm $\bar{3}m$ (225)^32^ precipitate from multi-component alloys generally contain Cr, Fe, Mn and Mo additions. For simplicity, only its main-composition Cr and Fe are considered here due to the lower content of Mn and Mo atoms in chromium steels. Regarding the measurements of the concentrations of substitutional solute atoms Fe and Cr in carbides Fe_19.51_Cr_3.49_C_6_ in Fig. 1a, Au replica specimens were prepared to avoid the influence of the matrix on the measured concentrations using a transmission electron microscopy (TEM) equipped with an energy-dispersive spectroscopy system. More than 70 isolated particles of M_23_C_6_ were examined. The average d-spacing of the {220} planes was measured to be 0.361 nm, and the lattice parameter of M_23_C_6_ carbide was determined to be 1.021 nm by SAED analysis, as shown in Fig. 1b. The experimental crystal lattice of Fe_20_Cr_3_C_6_ was 10.21 ± 0.32 Å at 823 K. The experimental d-spacing is within the error range of the theoretical value of 10.41 Å^25^. Alloy carbides M_23_C_6_ were remarkably promoted with the presence of 12 T magnetic field, whereas only M_3_C and M_2_C carbides (Fig. 1c,d) precipitated without the external field (Table 1).

**Table 1:** The carbides in 2.25Cr-Mo steel at 823 K without and with the application of 12 T magnetic field. M = Fe, Cr.

Tempering temperature, K	0 T	12 T
823	M_2_C, M_3_C	M_2_C, M_3_C, M_7_C_3_, M_23_C_6_ (Fe_19.51_Cr_3.49_C_6_)

The majority of thermodynamics analyses of precipitation stability under the magnetic field focus either purely on the absolute temperature^10,24^ or on the temperature dependence with lower magnetic field (<2 Tesla)^33^. On the one side, although the magnetic free energy at 0 K is most often considered in the analysis of carbide stability^10,24^, only a qualitative explanation was employed to explain the magnetic-field-induced precipitation behaviours. On the other side, in fact, the magnetic free energy under the low-strength field is much lower than that under the higher field (see Supplementary Figs S1 and S2), which means the high field significantly affects the thermodynamic properties and thus favours altering the precipitation stability.

### Magnetism characteristics

Before exploring the magnetic-field-induced stability, we first systematically examine the magnetism characteristics of alloy carbides M_23_C_6_ in two stages: first, we choose the most effective compound Fe_20_Cr_3_C_6_ which are taken as an example to analyse the detailed framework structure and stabilizing atoms for the complex Fe_23-x_Cr_x_C_6_; secondly, we investigate the interactions among the antiparallel spin of chromium, Fe-C bond distances and various Wyckoff sites of iron atoms on further clarification of the magnetic influence on stability at absolute temperature.

**Figure 2:**
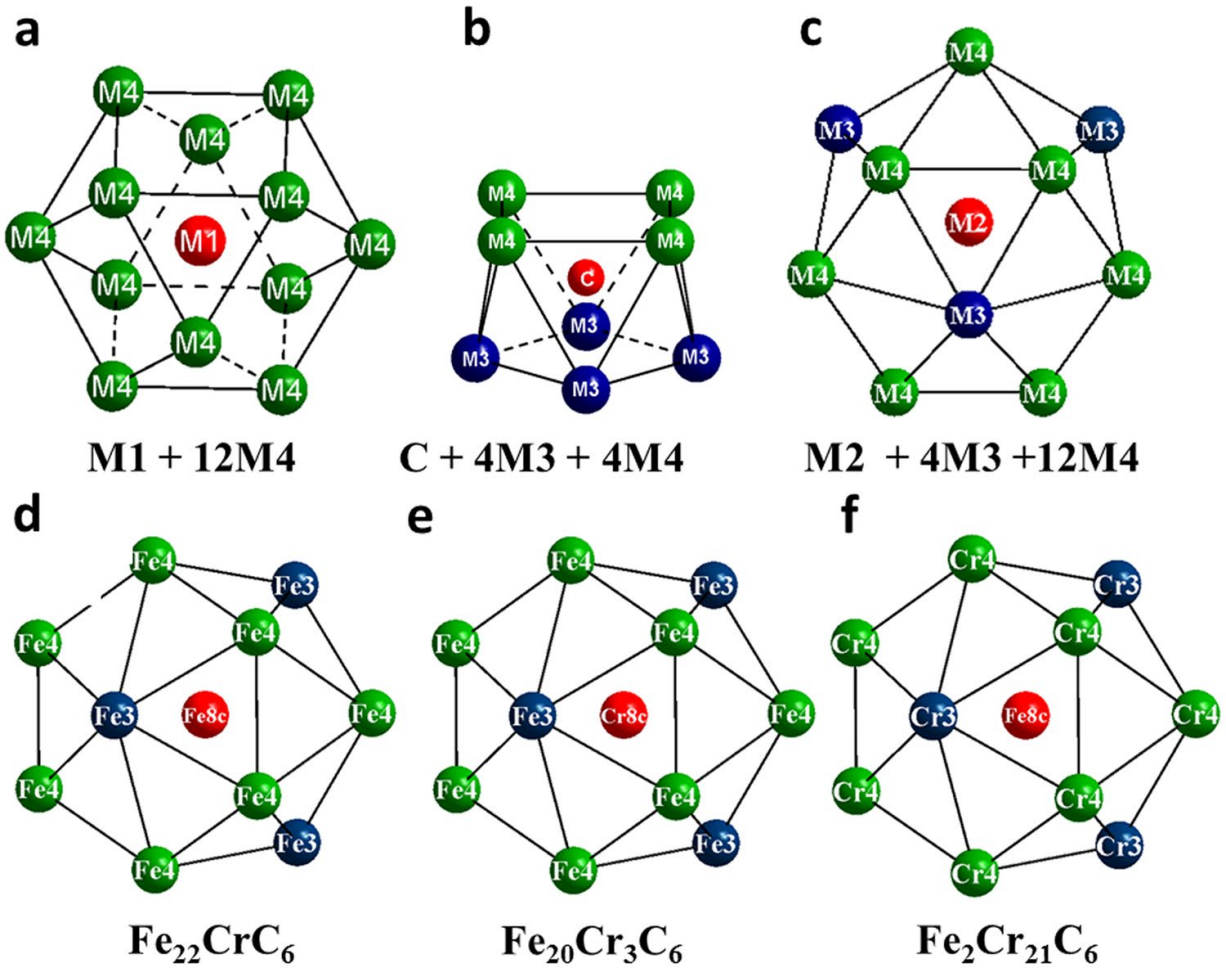
Framework for alloy carbide Fe_23-x_Cr_x_C_6_. (**a**–**c**) The structure of M_23_C_6_, which is composed of the frame (M4 (48h) and M3 (32f)) and stabilizers (M2 (8c), M1 (4a) and C (24e)). The red atom in the centre represents the 8c, 4a and 24e Wyckoff sites. The green and blue atoms represent the frame composed of the 48h and 32f Wyckoff sites for the M4 and M3 atoms, respectively. (**d**–**f**) The framework of the M4, M3 and M2 sites. The red atom in the centre represents the 8c Wyckoff position, and the green and blue atoms represent the 48h and 32f Wyckoff sites for Fe and Cr atoms, respectively.

In the first stage, as prerequisites we employed first-principles (see Methods) to calculate magnetic moment for the experimental composition (Fe_19.51_Cr_3.49_C_6_) which is approximately the same as Fe_20_Cr_3_C_6_. Similar to iron carbide Fe_23_C_6_^4^, alloy carbide Fe_20_Cr_3_C_6_ has 116 atoms and five crystallographically different atomic sites in a conventional unit cell, as shown in Table 2: Cr1 at 4a (origin symmetry, m $\bar{3}$ m), Cr2 at 8c (site symmetry $\bar{4}$ 3m), Fe3 at 32f (site symmetry .3 m) and Fe4 at 48h sites (local symmetry m.m2). The carbon atoms are at 24e (symmetry 4 m.m). Cr1 and Cr2 atoms are surrounded by Fe3 and Fe4 atoms. Thirty-two Fe3 and forty-eight Fe4 atoms form two groups of networks^4^. The two networks are strongly linked, and they together form the frame of the lattice of alloy carbide Fe_20_Cr_3_C_6_. Therefore, the structure of M_23_C_6_ can be considered to consist of a frame (M4 (48h) and M3 (32f)) and stabilizers (M2 (8c), M1 (4a) and C (24e)), as shown in Fig. 2a–c. Twelve nearest-neighbour M4 atoms surround one M1 atom (Fig. 2a). Likewise, four M3 and four M4 atoms form a cavity, the centre of which is occupied by a C atom, as shown in Fig. 2b. Each M2 atom has four nearest-neighbour M3 atoms and twelve M4 atoms (Fig. 2c). The coordination numbers (CNs) of the Fe atoms at the M3 and M4 sites follows the trend Fe4 (CN = 9 + 5) > Fe3 (CN = 9 + 4) for alloy carbide Fe_20_Cr_3_C_6_. Here, the first coordination number of the neighbours represents Fe-Fe coordination and the second Fe-Cr or Fe-C coordination. This CN order is consistent with the order of the magnetic moments, as shown in Table 2.

**Table 2:** Calculated the nearest neighbor bonds, magnetic moments and CNs for Fe_20_Cr_3_C_6_.

Atom	Site	Wyckoff position	Bonds	M (*μ*_*B*_)	CNs
Cr1	4a	0, 0, 0	−Fe4: 2.581 (×12)	−0.44	12
Cr2	8c	0.25, 0.25, 0.25	−Fe4: 2.876 (×12) −Fe3: 2.485 (×4)	−0.43	16
Fe3	32f	0.386, 0.386, 0.386	−Fe4: 2.623 (×6) −Fe3: 2.405 (×3) −Cr2: 2.485 (×1) −C: 2.133 (×3)	1.31	13
Fe4	48h	0, 0.173, 0.173	−Fe4: 2.581 (×5) −Fe3: 2.623 (×4) −Cr1: 2.581 (×1) −Cr2: 2.876 (×2) −C: 2.062 (×2)	1.56	14
C	24e	0.264, 0, 0	−Fe4: 2.062 (×4) −Fe3: 2.133 (×4)	−0.075	8

Based on the structural framework presented above, we also examine how the interaction between the atoms Fe, Cr and C play important roles in the magnetism characteristics (see Supplementary Fig. S3). The magnetizations per unit cell obtained from the first-principles calculations in Fe_23-x_Cr_x_C_6_ carbides are plotted as functions of the number of electrons per atom (e/a) (see Supplementary Fig. S4). The magnetization increases with the Fe concentration. In the structure of Fe_22_CrC_6_, Fe, Cr and C atoms occupy the Wyckoff positions 48h/32f/8c, 4a and 24e, respectively (Fig. 2d). Focusing on Fe, which is expected to contribute mainly to the magnetic properties of the system, the 48h/32f/8c sites form a three-dimensional framework. Therefore, we have compared ferromagnetic Fe_20_Cr_3_C_6_ and Fe_2_Cr_21_C_6_ with different Cr substitutions by Fe atoms in the same framework (Fig. 2e,f). In contrast to the network in Fe_22_CrC_6_, the 8c Wyckoff positions are occupied by Cr atoms for Fe_20_Cr_3_C_6_, as shown in Fig. 2(e). Similar to Fe_20_Cr_3_C_6_, the framework of Fe_2_Cr_21_C_6_ can be obtained by substituting Fe3 and Fe4 atoms with Cr in Fe_22_CrC_6_ (Fig. 2f). The magnetic moment is found to equal approximately 2.11 *μ*_*B*_ per three-dimensional framework in Fig. 2(d) by considering the contributions of the Fe atoms in the 48h (×12), 32f (×4) and 8c (×1) sites. Different from Fe_22_CrC_6_, the magnetic moments in the networks of Fe_20_Cr_3_C_6_ and Fe_2_Cr_21_C_6_ (Fig. 2e,f) resulted from the cooperation of Fe and Cr atoms. The average magnetic moment of a Cr atom in Fe_20_Cr_3_C_6_ is −0.43 *μ*_*B*_, as shown in Table 2. The local magnetic moments of Cr are aligned anti-parallel to those of the Fe atoms^34^. The total magnetization of bcc Fe is reduced by Cr addition as a result of the antiparallel spin interaction, with the opposite effect for solutes that couple ferromagnetically^35^. It is apparent that more Cr in Fe_23-x_Cr_x_C_6_ results in a smaller magnetic moment. Correspondingly, the average magnetic moments in the frameworks of Fe_22_CrC_6_, Fe_20_Cr_3_C_6_ and Fe_2_Cr_21_C_6_ are 2.03, 1.38 and 0.063 *μ*_*B*_. This result essentially coincides with the assessment that higher Fe content greatly increases the magnetic moments of alloy carbides^14^.

Additionally, the nearest-neighbour Fe-C bond length in the 32f position at absolute zero (Table 2) for Fe_20_Cr_3_C_6_ is approximately 2.13 Å, which is slightly larger than that in the 48h site. Moreover, the shorter bond length in 48h corresponds to the larger local magnetic moment of approximately 1.56 *μ*_*B*_, compared to 1.31 *μ*_*B*_ for Fe in 32f. This indicates that the ferromagnetism increases with the reduction of Fe-C bond lengths upon the removal of a Cr atom. This agrees with the conventional wisdom that the strong hybridization of the Fe-C interaction reduces the local moment of the Fe atom^4,5,36^. The d-band polarization determines the Fe magnetic moment, whereas the magnetic moment of a carbon atom originates principally from the delocalized Fe d states that overflow from the spheres^34,37^.

**Figure 3:**
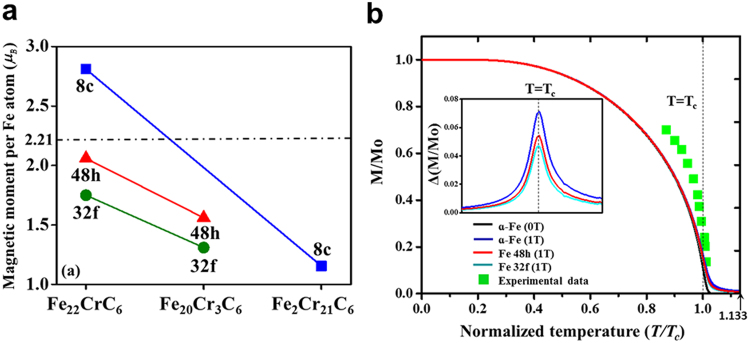
(**a**) Magnetic moments of Fe atoms calculated from first-principles at absolute zero in Fe, Fe_22_CrC_6_, Fe_20_Cr_3_C_6_ and Fe_2_Cr_21_C_6_. Furthermore, the magnetic moments in the 48h and 32f Wyckoff positions of Fe_20_Cr_3_C_6_ were obtained using first principles and employing an experimental crystal lattice with a d-spacing of 10.21 Å. Note that the experimental magnetic moment (dotted line) of pure Fe was considered as the reference^39^. (**b**) Variation of reduced magnetization for Fe atoms in 48h and 32f sites in Fe_20_Cr_3_C_6_ with temperature and magnetic field dependences in comparison with the corresponding experimental data (green square)^40^. Note that the saturation magnetization of Fe_20_Cr_3_C_6_ at 0 K was obtained by first-principles calculation, employing the experimental crystal lattice with a spacing of 10.21 Å at 823 K for Fe_19.51_Cr_3.49_C_6_. The insert shows the magnetic differences (Δ (M/Mo)) by comparing the pure Fe (0 Tesla) with pure Fe (1 Tesla) and Fe atoms in 48h and 32f sites (1 Tesla).

A critical issue for the understanding the dependence of the precipitation behaviours on the external field is the magnetic moment per Fe atom. It is known that the thermodynamic stability of carbides can be changed by the positon of Fe atoms^15,17,21^. To examine the influence of magnetism on the stability of the ferromagnetic phase M_23_C_6_, we performed calculations of the Fe average magnetic moments at Wyckoff sites (48h, 32f or 8c) in Fe, Fe_22_CrC_6_, Fe_20_Cr_3_C_6_ and Fe_2_Cr_21_C_6_ from first-principles (Fig. 3a**)**. The increasing Cr content corresponds to the decrease in the Fe magnetic moment per Fe atom in Fe_22_CrC_6_, Fe_20_Cr_3_C_6_ and Fe_2_Cr_21_C_6_. The magnetic moments per Fe atom in Fe_22_CrC_6_, Fe_20_Cr_3_C_6_ and Fe_2_Cr_21_C_6_ were smaller than that of pure Fe^38^. However, the value for the 8c site in Fe_22_CrC_6_ deviated markedly from those for other compositions and other types of site, as shown in Fig. 3a. Fe2 is coordinated by twelve Fe4 and four Fe3 in Fe_22_CrC_6_ (Fig. 2d), whereas Fe2 is coordinated by twelve Cr4 and four Cr3 in Fe_2_Cr_21_C_6_ (Fig. 2f). Therefore, the magnetic moment of Fe in Fe_2_Cr_21_C_6_ is reduced by the increasing number of negative magnetic moment of Cr atoms. Comparing the 48h site in Fe_20_Cr_3_C_6_ with that in Fe_22_CrC_6_, the increased Cr content corresponds to a lower magnetic moment value. Similar to the 48h site, the 32f site in Fe_22_CrC_6_ is surrounded by six Fe4, three Fe3, three C and one Fe2, whereas the 32f site in Fe_20_Cr_3_C_6_ is located in the centre of six Fe4, three Fe3, three C and one Cr2. The substitution of antiparallel spin of Cr neighbours into the 48h/32f/8c sites of Fe atom causes smaller magnetic moments.

However, studies have focused so far on properties at T = 0 K and 0 Pa for different structures and compositions only and do not capture temperature and external field effects, thus giving rise to the possibility of a gap between the theoretical and real experimental results. Furthermore, carbide stability at the ground state (0 K and 0 Pa) has been investigated by means of the formation energy. It is found that the stability of M_23_C_6_ at absolute zero is the lowest compared with M_7_C_3_, M_3_C and M_2_C (see Supplementary Fig. S5). However, experimentally, the effect of magnetic field strength on M_23_C_6_ is much more remarkable than on M_7_C_3_, M_3_C and M_2_C at an intermediate temperature during the tempering of 2.25Cr-Mo alloy. Therefore, we further consider the coupling interaction of the temperature and external field and then discuss the accuracy of the variation of the field-induced magnetism.

### Magnetic moment dependence on external field and temperature

Having verified the influence of structure and composition on the magnetic moment at absolute zero, we now focus on accurately describing the magnetism related to the external field and temperature according to the improved Weiss theory (see equations (2–4) in Supplementary). The dependence on the external field (1 Tesla) and temperature (approximately the Curie temperature) of the magnetization, in comparison with the corresponding experimental data (green square)^39^, is shown in Fig. 3b. The calculated results fail to fully quantitatively describe the shape of the experimental results; this is because the experiments were performed on multiphase samples that contained mixture phases. Nevertheless, as shown in Fig. 3b, qualitatively, the magnetization under 1 Tesla in Fe is reproduced well.

One interesting result is that polarized neutron scattering experiments without the external field^40^ and theoretical predictions based on the extended dynamic spin-fluctuation theory^41^ have verified that there is still a substantial magnetic moment in the paramagnetic phase above Tc. This means that there are still exchange interactions between atoms at different sites^42^. The exchange interaction causes the splitting in the density of states of the *e*_*g*_ orbits near the Fermi level^43^. The more incoherent *e*_*g*_ and more itinerant *t*_*2g*_ electrons accompany the formation of local magnetic moments in paramagnetic α-Fe^43,44^. The magnetic moments at atomic sites i and j increase with magnetic exchange coupling. The magnetic exchange coupling and the spin quantum number entirely determine the Heisenberg Hamiltonian, and then, the magnetic free energy is derived using many-body theory^45^.

When the magnetic field is applied, the direction of ordered moments is determined by the competition among the exchange interaction, crystal field effect^46^ and external field. To better understand the role of the magnetic-field-induced magnetization, we focus on the results for Fe in three different states. One is α-Fe, and the others are in Wyckoff sites 48h or 32f in alloy carbide Fe_20_Cr_3_C_6_. As seen in Fig. 3b, up to 0.95 Tc, three curves show almost identical tendencies, in contrast to the results in the absence of an external field. This means that the effects of the external magnetic field on the ferromagnetism of three different Fe states are assumed to be identical. Going around Tc, the magnetic-field-induced magnetization increases to a higher level for the 48h site than for the 32f site and is highest for α-Fe. This is a direct consequence of the increase in the exchange coupling in the presence of the applied magnetic field. The magnetic-field-induced exchange coupling causes the parallel magnetic moment to increase, which increases the magnetic moment. The insert in Fig. 3b shows that the largest discrepancies are found around Tc, with the sequence α-Fe > 48h > 32f. This is because a noticeable amount of magnetic energy in α-Fe is still stored in the magnetic short-range order, whereas the smaller magnetic-field-induced magnetization in 32f due to the Wyckoff position corresponds to the storage of a smaller amount of magnetic energy. The storage of less magnetic energy in 32f in the paramagnetic state corresponds with weaker magnetic exchange coupling. A higher temperature extension (T ≥ 1.133Tc) at the α-γ structure phase transition point, driven by the magnetic correlation energy^47,48^ and external field, may change the transition point. Nevertheless, it is beyond the scope of this paper.

**Figure 4:**
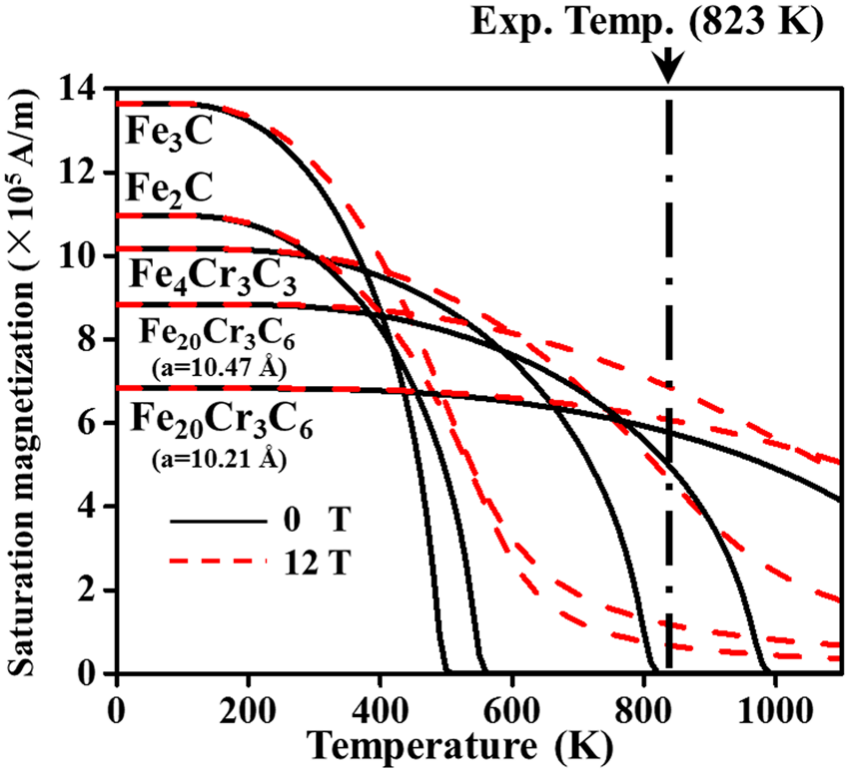
Dependence of the simulated saturation magnetizations of alloy carbides Fe_20_Cr_3_C_6_, Fe_4_Cr_3_C_3_, Fe_3_C and Fe_2_C on temperature and magnetic field strengths. The saturation magnetization curve of Fe_20_Cr_3_C_6_ (a = 10.21 Å) was obtained by improved Weiss molecular field theory, employing the experimental crystal lattice with a spacing of 10.21 Å at 823 K for Fe_19.51_Cr_3.49_C_6_. Note that the Fe_20_Cr_3_C_6_ (a = 10.47 Å) curve originates from the crystal lattice with a spacing of 10.47 Å that was obtained from the theoretical data in the first-principles calculation.

The magnetization of an alloy carbide is determined by the total contribution of the magnetic moments Fe, Cr and C. Furthermore, the magnetization of pure Fe^18,49^ or alloy carbides^14,24^ increases with the external field strength and magnetic atom content. Figure 4 shows that lower temperature, higher external field and higher Fe content increase the ferromagnetism of alloy carbides M_23_C_6_, M_7_C_3_, M_3_C and M_2_C. At 823 K, the magnetic-field-induced magnetization follows the sequence Fe_20_Cr_3_C_6_ (Cal.) > Fe_20_Cr_3_C_6_ (Exp.) > Fe_4_Cr_3_C_3_ > Fe_3_C > Fe_2_C. Based on the above experimental results, the combined effect of the structure, composition, temperature, and external field is closely linked to the precipitation of the alloy carbide. The magnetic-field-induced precipitation behaviours are determined by not only the magnetic effect but also the thermal influence. Complete understanding of the magnetic and thermal effects on alloy carbide stability is utilized to develop a profound and proper interpretation of the influence mechanism of the external field on the precipitation behaviours.

### Thermal contributions for four types of carbides

The thermodynamic software MTDATA is used from the UK’s National Physical Laboratory^14,23^ to obtain the thermal contributions for carbides in steels, such as the composition of equilibrium phases, the diffusion of interstitial and substitutional atoms or the Gibbs free energy. In principle, the thermal contribution corresponds to the minimized Gibbs free energy of the system when the precipitation occurs by diffusion of both interstitial and substitutional atoms. In the present work, Δ*G*_th_(*S*, *X*, *T*) of alloy carbides were calculated with version 1.0 using the TCAB database. Ferrite and alloy carbides are identified as the parent and product phases, respectively, to coincide with the experimental results^24^. Thermal Gibbs free energies were calculated for four alloy carbides: Fe_20_Cr_3_C_6_, Fe_4_Cr_3_C_3_, Fe_3_C and Fe_2_C. Several discontinuities were found during the simulation of the phase transformation, but they hardly have influence on the determining factor and will not be considered in this study.

### Magnetic Gibbs free energy

**Figure 5:**
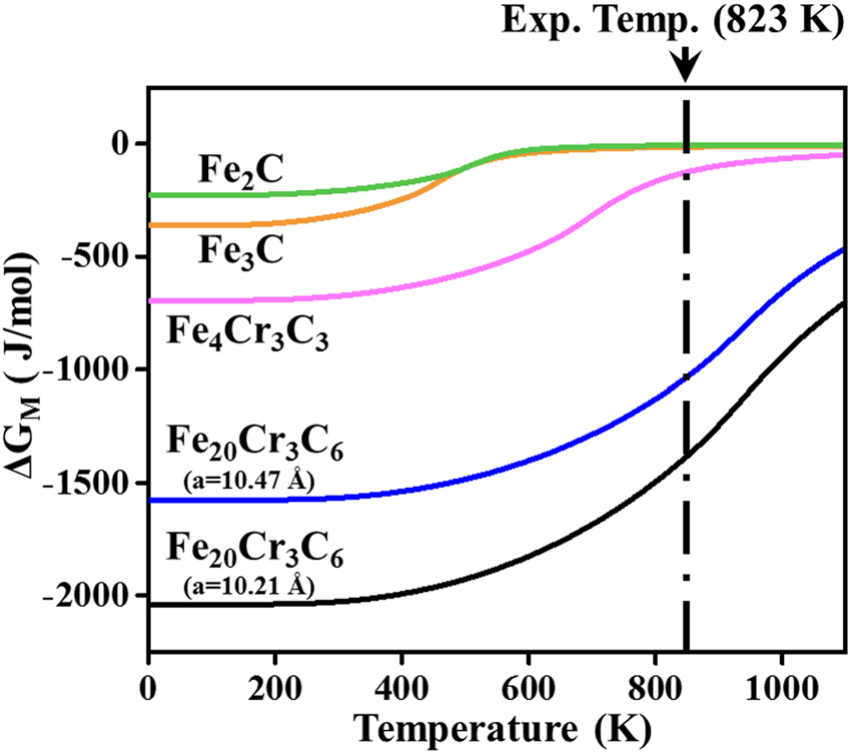
Calculated magnetic energy changes in the presence of high magnetic field for alloy carbides M_23_C_6_, M_7_C_3_ M_3_C and M_2_C. The magnetic free energy of Fe_20_Cr_3_C_6_ (a = 10.21 Å) was obtained by equation (1) in the Supplementary information by employing the experimental crystal lattice with a spacing of 10.21 Å at 823 K. Note that the Fe_20_Cr_3_C_6_ (a = 10.47 Å) curve originates from the crystal lattice with a spacing of 10.47 Å that was obtained from the theoretical data in the first-principles calculation. Due to the large energy change in the theoretical predication, the benchmarks are subject to an uncertainty that will hopefully be reduced in the future by progress in materials synthesis and experimental setups.

The magnetic Gibbs free energy under the external field (see equation (1) in Supplementary), which is closely related to the stability, is lowered according to the structure, composition, magnetization and magnetic field strength. The magnetic-field-induced Gibbs free energy change of Fe_20_Cr_3_C_6_ was higher than those of the other alloy carbides, as shown in Fig. 5. Additionally, experimental results on Mo-containing alloy have verified that the external field has an obvious influence on the Fe concentrations of precipitates^11^. However, the magnetic contribution is not necessarily the determining factor for the magnetic-field-induced carbide stability. The stability depends on a comprehensive assessment that considers the combined effects of magnetism, temperature, and external field dependences.

**Figure 6:**
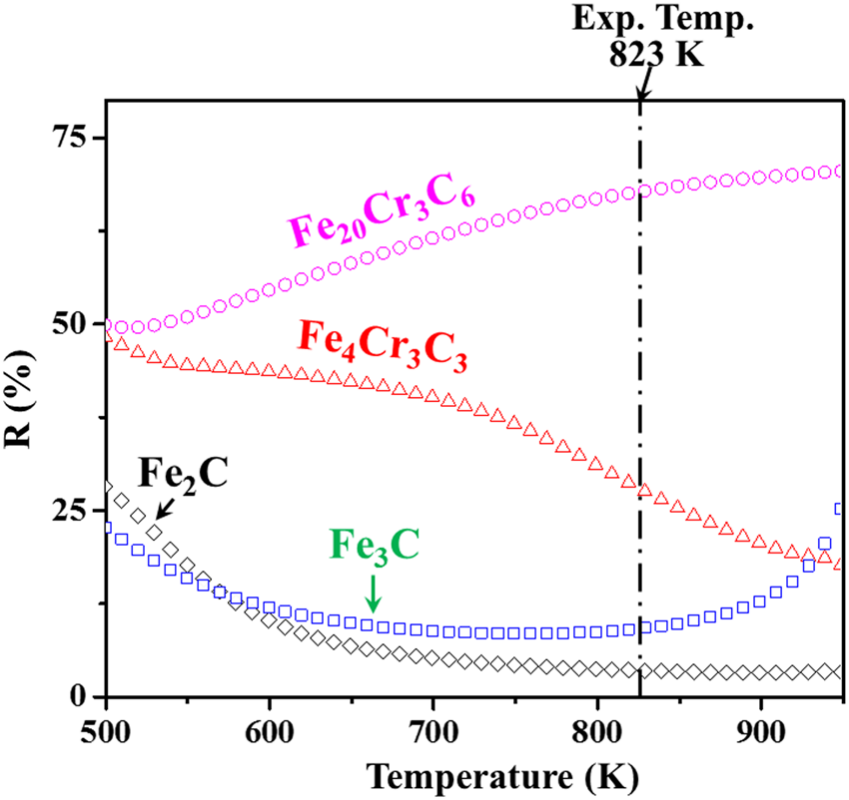
The controlling factor *R* at 12 T high magnetic field as a function of temperature, including the carbide type’s contributions. The dash-dot line shows the experimental temperature.

To facilitate direct comparison with experimentally accessible quantities, the major controlling factor *R* (see equation (6) in Supplementary) is computed to characterize the magnetic-field-induced precipitation behaviours. The magnetic and thermal Gibbs free energies both determine the *R* value under 12 T magnetic field. It is of interest to observe in Fig. 6 that *R* > 50% for M_23_C_6_, whereas *R* < 50% for the other carbides, namely, M_7_C_3_, M_3_C and M_2_C. This indicates that the magnetic free energy contributions to the total free energy are significantly different. The magnetic field has a predominant effect over the whole temperature range for M_23_C_6_, whereas thermal effects are much more significant for M_7_C_3_, M_3_C and M_2_C. When the temperature reaches 823 K, the Gibbs free energy change of Fe_20_Cr_3_C_6_ drops below those of Fe_4_Cr_3_C_3_, Fe_3_C and Fe_2_C under a 12 T magnetic field. The calculated thermodynamic properties induced by the high magnetic field show excellent agreement with the available experimental data in Table 1 and reveal the necessity of considering the fourfold coupling interaction of structure, composition, temperature and external magnetic field.

## Discussion

In the present work, we have discovered experimentally that an external high magnetic field effectively promotes the precipitation of M_23_C_6_ carbides at intermediate temperature in quenched and tempered specimens. Meanwhile we theoretically construct an integrated method, which combines first-principles calculation with the improved Weiss molecular field theory and thermodynamic software, to examine the origins of the thermodynamics stability enhancement that results from high external field. We proposed three lattices structures at absolute zero in order to elucidate the essence of magnetism change during the substitution of more Cr atom into pure iron carbide Fe_23_C_6_. Our results show that magnetic characteristics are derived from the interactions between the various Wyckoff sites of iron atoms, antiparallel spin of chromium and Fe-C bond distances. Moreover, around Curie temperature, the magnetic-field-induced magnetization increases from 32f site over 48h and is highest for α-Fe. This is a direct consequence of the fact that the exchange coupling is increased with the presence of the applied magnetic field. We have proven theoretically that high magnetic field has a predominant contribution for M_23_C_6_ whereas the thermal effect is a determining factor for M_7_C_3_, M_3_C and M_2_C.

These discoveries contribute to a better understanding of the creep-resistant property in the condition of high temperature and strong magnetic field and are expected to substantially broaden the development of ITER project in fusion energy. In a broader perspective, our observation of the magnetic-field-induced behaviours will certainly push to develop magnetic phase transformation theory for the family tree of Fe-based materials that contain the elements Mo, V, W, Ti, etc. From an instrumental point of view, this work paves the way for the investigation on the different magnetism for the alloy carbides systems under high magnetic field.

## Methods

### First-principles calculation of different alloy carbides

All magnetic moment calculations were carried out using the first-principles Vienna Ab initio Simulation Program, which employs density-functional theory^50,51^ within the projector-augmented wave (PAW) method^52,53^. The generalized gradient approximation (GGA) was employed for the exchange and correlation energy terms since the GGA approximation describes spin-polarized Fe better than the local (spin-polarized) density approximation^54,55^. The PAW potentials for iron, chromium and carbon include the 3d^7^4s^1^ and 3d^5^4s^1^ orbitals, as well as 2s^2^2p^2^ valence states. Furthermore, considering the influence of the electronic correlation inside the atomic d-shell of Fe, LSDA^35^ and GGA+U (Hubbard U = 2.2 eV) have been developed to describe the 3d impurities^4,56^. However, the results obtained using these methods have significant discrepancies with experimental data related to the stability of iron and iron-rich transition-metal alloys^4^. Therefore, in the present work, GGA was chosen for the magnetic moment calculations for alloy carbides.

The cut-off energy of the wave functions was 500 eV for the 3d transition metal carbides (including M_23_C_6_, M_7_C_3_, M_3_C and M_2_C). Reciprocal space integrations were carried out using dense k-meshes, e.g., 8 × 8 × 8 (20 k-point) to 12 × 12 × 12 (56 k-point) grids, in the irreducible Brillouin zone of the alloy carbides M_23_C_6_ using the Monkhorst and Pack method^57^, while 7 × 5 × 7 (48 k-point), 7 × 7 × 9 (80 k-point), 11 × 11 × 9 (80 k-point), 9 × 9 × 9 (35 k-point), 7 × 7 × 7 (21 k-point) and 7 × 7 × 7 (20 k-point) grids were used for Fe_3_C, η-Fe_2_C, ξ-Fe_2_C, α-Fe, C and Cr respectively. All calculations were spin-polarized to allow us to study magnetism in carbides. The tests on the k-meshes and cut-off energies showed good convergence (1 meV/atom).

## Electronic supplementary material

Supplementary Information
