# Author Correction: Magnetism and high magnetic-field-induced stability of alloy carbides in Fe-based materials

**DOI:** 10.1038/s41598-018-25978-5

**Published:** 2018-05-15

**Authors:** T. P. Hou, K. M. Wu, W. M. Liu, M. J. Peet, C. N. Hulme-Smith, L. Guo, L. Zhuang

**Affiliations:** 1 The State Key Laboratory for Refractories and Metallurgy, Hubei Province Key Laboratory of Systems Science in Metallurgical Process, International Research Institute for Steel Technology, Wuhan University of Science and Technology, Wuhan, 430081 China; 2 Beijing National Laboratory for Condensed Matter Physics, Institute of Physics, Chinese Academy of Sciences, Beijing, 100190 China; 3 Department of Materials Science and Metallurgy, University of Cambridge, Cambridge, UK; 4 Materials department, Centre of Excellence for Advanced Materials, Dongguan, 523808 China; 5 Sun Yat-Sen University, Guangzhou, 510275 China

Correction to: *Scientific Reports* 10.1038/s41598-018-20910-3, published online 14 February 2018

In the Supplementary Information file published with this Article, L. Guo is incorrectly affiliated with ‘Department of Materials Science and Metallurgy, University of Cambridge, UK’. The correct affiliation is listed below:

‘Materials department, Centre of Excellence for Advanced Materials, Dongguan, 523808, China’.

