# Supplementary material for: Magnetism and high magnetic-field-induced stability of alloy carbides in Fe-based materials
Source: Sci Rep. 2018 Feb 14;8:3049. doi: 10.1038/s41598-018-20910-3 (PMC5813241; doi:10.1038/s41598-018-20910-3)
Supplement: Supplementary file 1 — Supplementary Information [file 41598_2018_20910_MOESM1_ESM.pdf]

Supplementary information for "Magnetism and high magnetic-field-induced stability of alloy carbides in Fe-based materials"

T. P. Hou,[1,*] K. M. Wu,[1,*] W. M. Liu [2], M. J. Peet,[3]

C. N. Hulme-Smith,[3] L. Guo[3] and L. Zhuang[4]

[1] *The State Key Laboratory for Refractories and Metallurgy, Hubei Province Key Laboratory of Systems Science in Metallurgical Process, International Research Institute for Steel Technology, Wuhan University of Science and Technology, Wuhan 430081, China*

[2] *Beijing National Laboratory for Condensed Matter Physics, Institute of Physics, Chinese Academy of Sciences, Beijing 100190, China*

[3] *Department of Materials Science and Metallurgy, University of Cambridge, UK*

[4] *Sun Yat-Sen University, Guangzhou 510275, China*

*Corresponding author. Tel.: +86 27 68862772; Fax: +86 27 68862606; *E-mail address*: houtingping@wust.edu.cn;

*Corresponding author. Tel.: +86 27 68862772; Fax: +86 27 68862606; *E-mail address*: wukaiming@wust.edu.cn;

Magnetic-field-induced stability

The magnetic Gibbs free energy change $\Delta G_M(S, X, T, B)$ in equation (1), as a function of the structure *S*, composition *X*, temperature *T* and external field *B*, which is related to the stability, is lowered with the increase of the magnetization *M* and external field strength.

$$\Delta G_M(S,X,T,B) = -\mu_0 \int_0^B \vec{M}(S,X,T,B) \cdot d\vec{B} \tag{1}$$

The improved Weiss molecular theory can be expressed using equation (2):

$$M(S,X,T,B) = Nm(S,X,T,B)B_j(a_j), \tag{2}$$

where *N*, *m(S,X,T,B)* and *j* are the number of atoms per unit volume, the magnetic moment and quantum number, respectively. $B_j(\alpha_j)$ is the Brillouin function, which is defined as

$$B_j(\alpha_j) = \left\{ \frac{2j+1}{2j} cth \frac{(2j+1)a_j}{2j} - \frac{1}{2j} cth(\frac{a_j}{2j}) \right\}, \tag{3}$$

$$\alpha_j = \frac{n_B \mu_B B}{kT}, \tag{4}$$

where n_B, μ_B and k is the effective Bohr magneton number, Bohr magneton and the Boltzmann constant, respectively. The magnetization at absolute temperature is expressed $M_0 = Nm_0 \ B_j(a_j)$, where m_0 is the magnetic moment at 0 K which is calculated from first-principle calculation.

The magnetic free energy under low and high magnetic field strengths for iron and alloy carbides

Magnetic field effects on microstructure transformations in metallic materials were first reported in the middle of the last century by Smoluchowski and Turner [1]. Before the

1980s, magnetic field strength was limited to less than 2 Tesla; progress in powerful superconducting magnet generating techniques has made available high magnetic fields of strengths greater than 10 Tesla. The thermodynamic assessments under low- and high fields, which were performed according to the methodology presented in the section ``Magnetic-field-induced stability``, are shown in Supplementary **Figure S1**. The external field strength significantly affects the magnetic free energy over a large temperature window for pure Fe. As seen in **Figure S1**, the magnetic field contribution is the largest under the high magnetic field of 12 Tesla.

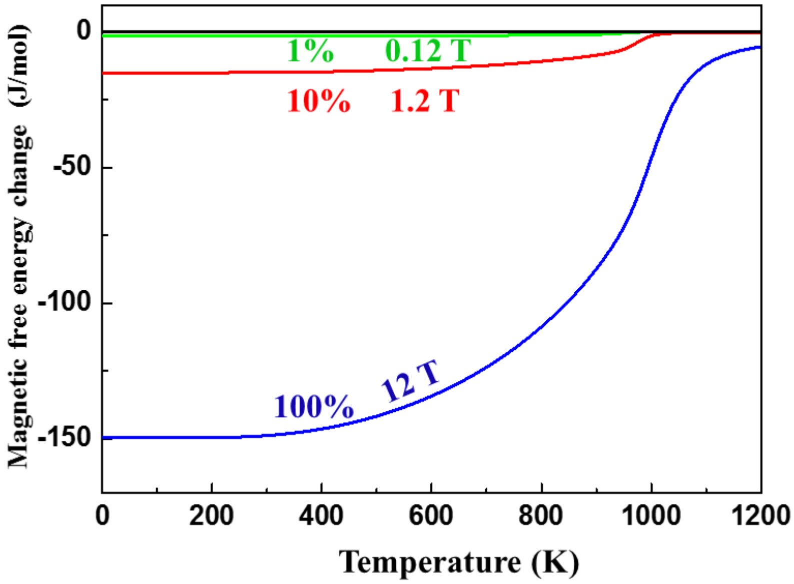

Figure S1. Comparison of magnetic free energy change between low (0.12, 1.2 Tesla) and high (12 Tesla) magnetic field strengths for pure Fe.

To further analyse the carbide`s performance in enhancing the magnetic-field-induced stability, we first consider the pure iron carbides, such as Fe_2C and Fe_3C. The results are shown in **Figure S2 and Table S1**. A negative magnetic free energy indicates that iron and alloy carbides are thermodynamically stable against temperature. This stability decreases

with increasing temperature. Therefore, we compare the magnetic free energies at 0 K only (**Table S1**). The magnetic free energy (T = 0 K) of iron carbide Fe_2C under the low-strength fields of 0.12 and 1.2 Tesla are approximately 99.1% and 90% larger, respectively, than that under the high magnetic field of 12 Tesla. Except for the iron carbide and pure iron, alloy carbides M_7C_3 and $M_{23}C_6$, which are the paramagnetic states to be borne in mind, are also greatly influenced by the high magnetic field of 12 Tesla. A closer inspection of $Fe_{20}Cr_3C_6$ shows that similar to iron carbides Fe_2C and Fe_3C, the low-strength fields (0.12 and 1.2 T) have almost no obvious effect on the magnetic free energy, while the high magnetic field greatly decrease the free energy value of 98.9% (**Table S1**). Therefore, this is a direct signal of the fact that an externally applied high magnetic field significantly affects their thermodynamic properties by altering the magnetic contribution to the total Gibbs free energy and thus changes their precipitation stability.

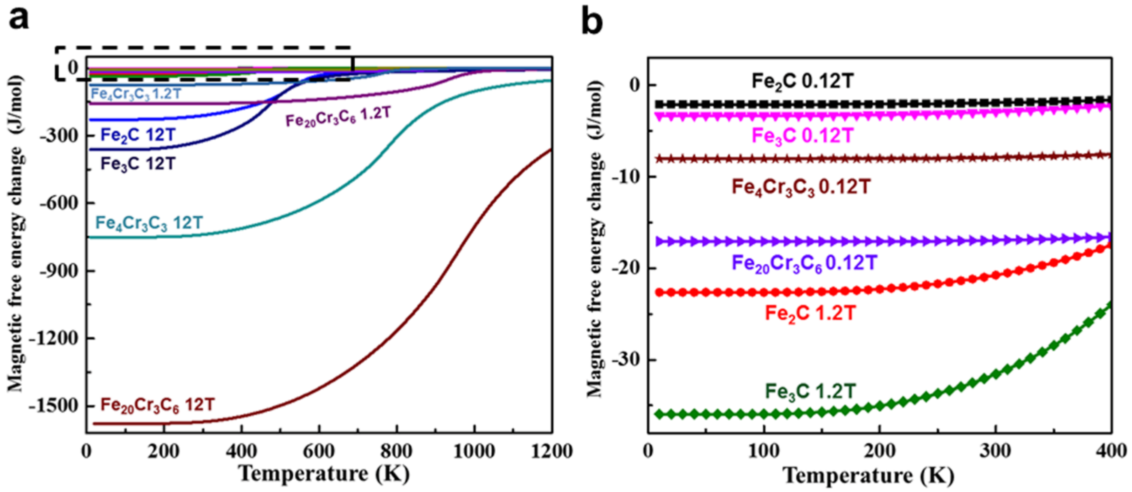

Figure S2. (a) Comparison of magnetic free energy change between iron carbides and alloy carbides in the present work. (b) Enlarged image from black dotted area in (a), showing a very small differences in the region close to zero for magnetic free energy.

Carbide types	0.12 T	1.2 T	12 T
Fe_2C	-2.09	-22.64	-228
Fe_3C	-3.32	-35.95	-362
$Fe_4Cr_3C_3$	-8.04	-74.8	-750
$Fe_{20}Cr_3C_6$	-17.07	-158.9	-1577

Table S1. Calculated magnetic free energy for iron carbides (Fe_2C and Fe_3C) and alloy carbides (M_7C_3 and $M_{23}C_6$) under the different magnetic field strengths at 0 K. M = Fe, Cr.

Major controlling factor

Thermal stability $\Delta G_{th}(S,X,T)$ is closely related to structure, chemical composition and temperature. The coupling of thermal free energy and magnetic free energy can provide a more accurate description and proper interpretation of magnetic-field-induced precipitation behaviours. When a magnetic field (12 T) is applied, the resulting free energy change $\Delta G_{total}(S,X,T,B)$ can be separated into two terms, as shown in equation (5): the thermal Gibbs free energy $\Delta G_{th}(S,X,T)$, which was determined using MTDATA software, and the magnetic Gibbs free energy $\Delta G_M(S,X,T,B)$.

$$\Delta G_{total}(S,X,T,B) = \Delta G_{th}(S,X,T) + \Delta G_M(S,X,T,B) \quad (5)$$

The major controlling factor R in equation (6) may be used to express the percentage ratio of the magnetic free energy change $\Delta G_M(S,X,T,B)$ to the total energy change $\Delta G_{total}(S,X,T,B)$.

$$R = \frac{\Delta G_M(S,X,T,B)}{\Delta G_{total}(S,X,T,B)} \times 100\% \quad (6)$$

Impact of composition on the magnetization

For the sake of comparison, we considered several possible configurations (**Figure S3**), according to the substitution of Fe by Cr such that the phase has the chemical composition $Fe_{23-x}Cr_xC_6$ (x = 0 ~ 23). No other unoccupied Wyckoff sites were considered because their interatomic spacing were too short. The average magnetic moments of the mixed systems are plotted in **Figure S4** as functions of the number of electrons per atom (e/a). The magnetization increases with the Fe concentration. The trend in magnetization of $Fe_{23-x}Cr_xC_6$ compounds is consistent with the calculation results [2-4].

Zero-temperature stability from formation enthalpy

First, the goal of this work is to study carbide stability at the ground state (0 K and 0 Pa) by means of the formation energy. The formation enthalpy per atom (ΔH) was described as a total energy difference between carbide $Fe_{23-x}Cr_xC_6$ and the constituent elements (α-Fe, graphite and chromium) in their stable states, as shown in the following equation (7)[2]:

$$\Delta H = (H\ (Fe_{23-x}Cr_xC_6) - ((23-x)\ H\ (Fe) + xH\ (Cr) + 6H(C)))\ /\ 29. \qquad (7)$$

At a temperature of 0 K and a pressure of 0 Pa, the enthalpy difference in the above equation is equal to the energy difference, which is $\Delta H = \Delta E$, when the zero-point vibration contribution is ignored [3]. ΔE is used to assess the stability of the carbide in the ground state. The vibration contribution is not considered because of the complexity of the polyatomic structure (for example, there are 116 atoms in the unit cell of $M_{23}C_6$). In **Figure S5,** we compare the ΔE values of four types of carbide, namely, $M_{23}C_6$, M_7C_3, M_3C and M_2C, and the results show that if only the thermal effect is considered, $M_{23}C_6$ is expected to be more stable than the other alloy carbides since it has the lowest formation energy. Furthermore, it is of interest to figure out that the stability of $Fe_{23-x}Cr_xC_6$ decreases with increasing x value. For $20 \leq x \leq 23$, which corresponds to all M1, M2 or M1+M2 occupations by iron,

the ΔE values are approximately -0.10 eV, and occupation of the M1 site by iron has the lowest value of -0.123 eV. This indicates that $FeCr_{22}C_6$ with an iron atom at the M1 site is the most stable carbide; this result is in accordance with References [4,5]. When at least one M3 site is filled with iron ($12 \le x \le 15$), it greatly destabilizes $Fe_{23-x}Cr_xC_6$. Finally, the occupation of more than one M4 site by iron ($0 \le x \le 11$) further decreases the stability of $Fe_{23-x}Cr_xC_6$.

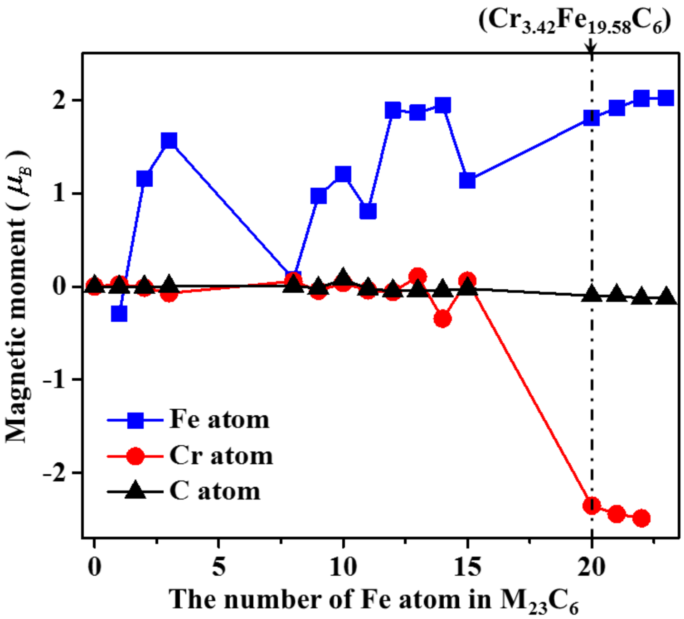

Figure S3. The calculated average magnetic moments per unit cell from the first principle calculation in $Fe_{23-x}Cr_xC_6$ carbides as a function of the number of electrons per atom. $M_{23}C_6$ includes the following composition: $Fe_{23}C_6$, $Fe_{22}CrC_6$, $Fe_{21}Cr_2C_6$, $Fe_{20}Cr_3C_6$, $Fe_{15}Cr_8C_6$, $Fe_{14}Cr_9C_6$, $Fe_{13}Cr_{10}C_6$, $Fe_{12}Cr_{11}C_6$, $Fe_{11}Cr_{12}C_6$, $Fe_{10}Cr_{13}C_6$, $Fe_9Cr_{14}C_6$, $Fe_8Cr_{15}C_6$, $Fe_3Cr_{20}C_6$, $Fe_2Cr_{21}C_6$ and $FeCr_{22}C_6$. The arrow represents the magnetic moments for different atoms in the experimentally measured carbide $Fe_{20}Cr_3C_6$ at 550°C. The magnetic moment data of $Fe_{20}Cr_3C_6$ was obtained by first principle calculation, employing the experimental crystal lattice 10.209 Å at 823 K.

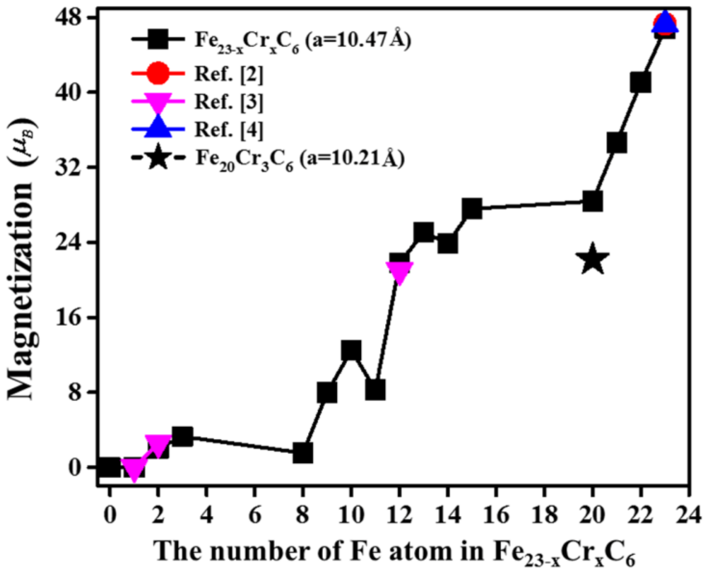

Figure S4. The calculated magnetization per unit cell from the first principle calculation in $Fe_{23-x}Cr_xC_6$ carbides as a function of the number of electrons per atom (e/a). The magnetization data of $Fe_{20}Cr_3C_6$ (pentagram) was obtained by first principle calculation, employing the experimental crystal lattice 10.21 Å at 823 K.

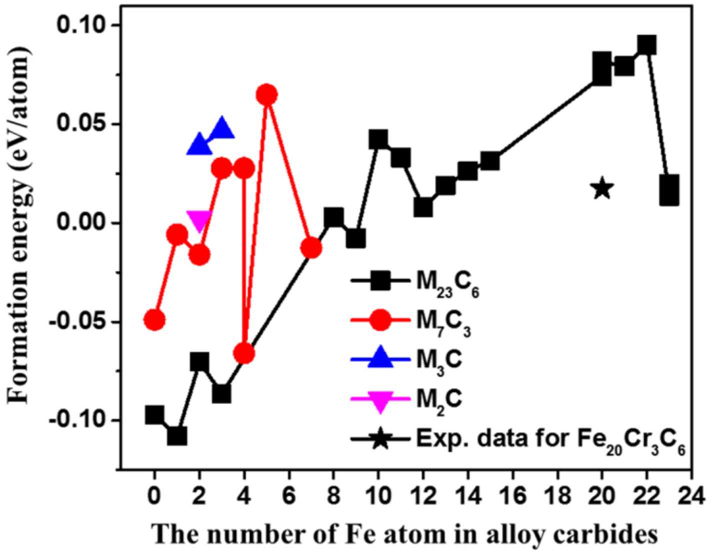

Figure S5. Formation energy of alloy carbide $M_{23}C_6$, M_7C_3, M_3C and M_2C as a function of Fe concentration. The formation energy data of $Fe_{20}Cr_3C_6$ (pentagram) was obtained by first principle calculation, employing the experimental crystal lattice 10.21 Å at 823 K.

Supplementary References

1. Smoluchowski R. & Turner R.W. Influence of magnetic field on recrystallization. *J. Appl. Phys*, **20**, 745 (1949).
2. Han, J. J., Wang C. P., Liu X. J., Wang Y. & Liu Zi-Kui. First-principles calculation of structural, mechanical, magnetic and thermodynamic properties for γ-$M_{23}C_6$ (M = Fe, Cr) compounds. *J. Phys.: Condens. Matter*. **24**, 505503 (2012).
3. Fang, C. M., van Huis M. A. & Zandbergen H.W. Stability, structure and electronic properties of γ-$Fe_{23}C_6$ from first-principles theory. *Acta Mater*. **58**, 2968-2977 (2010).
4. Fang, C. M., van Huis M. A. & Sluiter, M. H. F. Formation, structure and magnetism of the γ-$(Fe,M)_{23}C_6$ (M = Cr, Ni) phases: A first-principles study. *Acta Mater*. **103**, 273-279 (2016).
5. Medvedeva, N. I., Van Aken, D. C. & Medvedeva, J. E. Stability of binary and ternary $M_{23}C_6$ carbides from first principles. *Comp. Mater. Sci.* **96**, 159-164 (2015).
